# Supplementary material for: Molecular insights of exceptionally photostable electron acceptors for organic photovoltaics
Source: Nat Commun. 2021 May 24;12:3049. doi: 10.1038/s41467-021-23389-1 (PMC8144627; doi:10.1038/s41467-021-23389-1)
Supplement: Supplementary file 1 — Supplementary Information [file 41467_2021_23389_MOESM1_ESM.pdf]

Supplementary Methods

Molecular insights of exceptionally photostable electron acceptors for organic photovoltaics

Zhi-Xi Liu[1,+], Zhi-Peng Yu[1,5,+], Ziqiu Shen[1], Chengliang He[1], Tsz-Ki Lau[2], Zeng Chen[3], Haiming Zhu[3], Xinhui Lu[2], Zengqi Xie,[4] Hongzheng Chen[1], Chang-Zhi Li[1,*]

1. State Key Laboratory of Silicon Materials, MOE Key Laboratory of Macromolecular Synthesis and Functionalization, Department of Polymer Science and Engineering, Zhejiang University, Hangzhou 310027, P. R. China. E-mail: czli@zju.edu.cn
2. Department of Physics, The Chinese University of Hong Kong, New Territories, Hong Kong, P. R. China.
3. Department of Chemistry, Zhejiang University, Hangzhou 310027, P. R. China
4. State Key Laboratory of Luminescent Materials and Devices, School of Materials Science and Engineering, South China University of Technology, 381 Wushan Road, Guangzhou 510640, P. R. China
5. Present address: Institutes of Physical Science and Information Technology, Anhui University

+ These authors contributed equally.

Supplementary Methods

Materials All the chemicals including solvents, reagents, and catalysts were purchased from Sigma-Aldrich, Alfa Aesar chemical company, Sunatech Co., Ltd., and Derthon Optoelectronic Materials Science Technology Co LTD. Unless otherwise specified, such chemicals were used without any further purification. PBDB-TF, Y6 and the UV curing adhesive of epoxy resin LX803 were purchased from Solarmer Materials Inc.

Instrument. ^{1}H NMR, and ^{13}C NMR spectra were recorded on a Bruker Advance III (FT, DCH Cryoprobe, 400 MHz and 600 MHz for ^{1}H NMR and 126 MHz for ^{13}C NMR). MALDI-TOF MS spectra were obtained on a Walters MALDI-TOF Premier mass spectrometry. UV-vis absorption spectra were measured on a Shimadzu UV-2450 spectrophotometer. The PL spectra was measured by FluoroMax-4 HORIBA Jobin Yvon spectrofluorometer. Cyclic voltammetry (CV) was tested on a CHI600A electrochemical workstation with Pt disk, Pt plate, and standard calomel electrode (SCE) as working electrode, counter electrode, and reference electrode, respectively, in a 0.1 mol/L tetrabutylammonium hexafluorophosphate (Bu_4NPF_6) CH_2Cl_2 solution. The CV curves were recorded versus the potential of SCE, which was calibrated by the ferrocene-ferrocenium (F_c/F_c^+) redox couple (4.8 eV below the vacuum level). TEM images were performed on JEOL-1010 at 80 kV accelerating voltage in bright field mode. Time-resolved photoluminescence spectroscopy (TRPL) were detected by a home-setup microfluorescence system. All the samples were excited with a femtosecond laser (Light Conversion Pharos, 1030nm, ＜300fs, 1MHz) at 550nm.

TRPL decay kinetics were collected by a TCSPC module (PicoHarp 300) and a SPAD detector (IDQ, id100) with an instrument response function ~100 ps. GIWAXs measurements were carried out with a Xeuss 2.0 SAXS/WAXS laboratory beamline using a Cu X-ray source (8.05 keV, 1.54Å) and Pilatus3R 300K detector. The incidence angle is 0.2°.

Device fabrication: Polymer solar cells were fabricated on glass substrates commercially pre-coated with a layer of indium tin oxide (ITO) with the inverted structure of ITO/ZnO/SAM/PBDB-TF: Acceptors/MoO_3/Ag. Before fabrication, the substrates were cleaned by detergent, deionized water, acetone and isopropanol consecutively for every 15 min, and then treated in an ultraviolet ozone generator for 15 min. A thin layer of ZnO was spin-coated on clean ITO-coated glass substrates at 3500 rpm for 60 s and then annealed at 170 °C for 20 min. The SAM (1 mg mL^{-1} in tetrahydrofuran) were spin-coated onto ZnO layer at the rate of 4000 rpm for 40 s, followed by thermal annealing at 100 °C for 10 min. Later, substrates were washed by tetrahydrofuran at 4000 rpm for 40 s. The solution of PBDB-TF:NFAs in chlorobenzene (20 mg/ml, 1:1.2, w/w) was then spin-coated on the ETLs at 2500 rpm. Then the BHJ layers were annealed at 120°C under nitrogen atmosphere. After annealing, hole transporting layer MoO_3 (4 nm) was deposited on the active layers. Finally, silver (100 nm) was thermally evaporated through shadow masks.

***J-V* measurement:** The current density-voltage (*J-V*) curves of OSCs were measured with Keithley 2400, under AM 1.5G illumination at 100 mW/cm^2 irradiation using a Enli SS-F5-3A solar simulator, and the light intensity was calibrated with a standard Si

solar cell with KG5 filter (made by Enli Technology Co., Ltd., Taiwan, and calibrated report can be traced to NREL). A mask was used to define the active area (0.05979 cm^2) for the J-V measurements of devices. The EQE spectra were measured by a QE-R Solar Cell Spectral Response Measurement System (Enli Technology Co., Ltd., Taiwan).

TPV and CE: The *J–V* curves at varied light intensity, transient photo-voltage and charge extraction measurements were tested by PAIOS system. The cells were illuminated by a high-power white LED with a maximum intensity of 100 mW cm^{-2}.

Photostability tests: The photostability of films and encapsulated devices were measured with ISOS-L-3 setup (Infinity PV) in air (Humidity: 50 ± 5 %; Temperature: 40 ± 5 °C). The photostability tests were conducted under continuous 1 sun equivalent illumination provided by a metal halide lamp (PHILIPS MSR 1200HR) without UV-filter. Light intensity was calibrated by standard silicon solar cells.

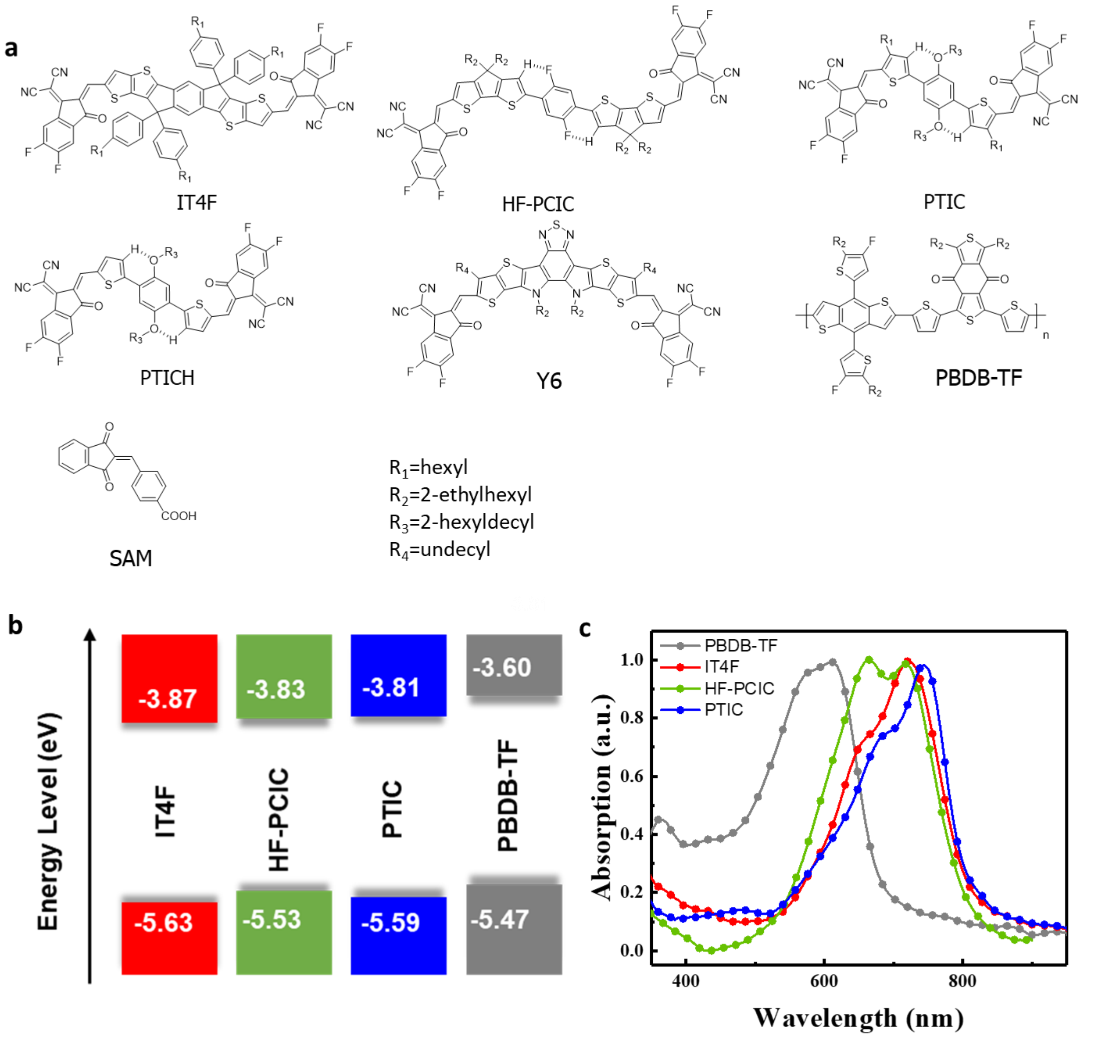

Supplementary Figure 1: a, Chemical structures of NFAs, donor and small molecule of self-assembled monolayers in the text. **b**, Energy levels for different active components **c**, UV–vis absorption of acceptors and donor in the films.

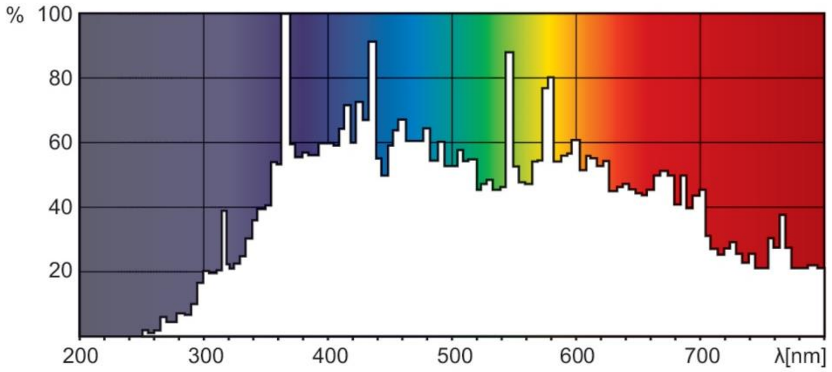

Supplementary Figure 2: Light spectrum of a metal halide lamp (PHILIPS MSR 1200HR) in ISOS-L-3 type photostability testing setup (Infinity PV).

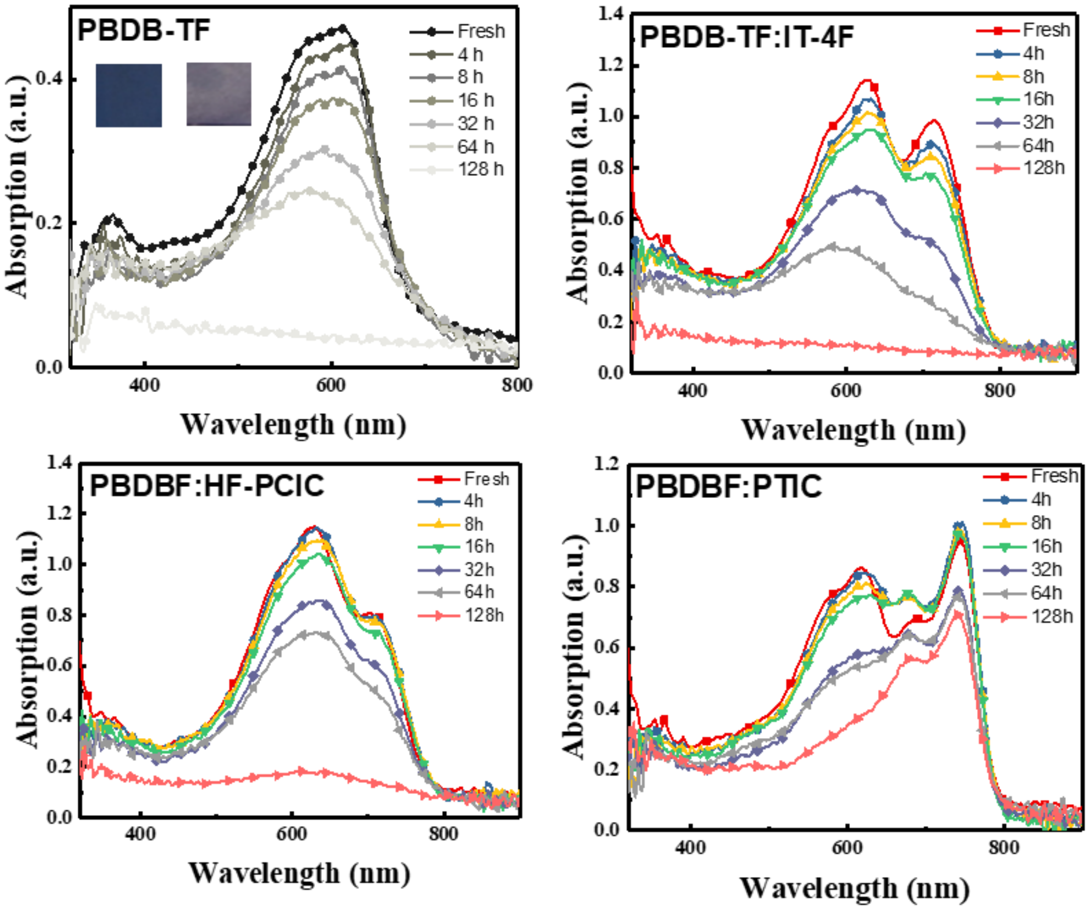

Supplementary Figure 3: UV–vis absorption spectra of neat polymer films and blend films with the thickness of ~ 110 nm (PBDB-TF of 92 nm, PBDB-TF: IT-4F of 108 nm, PBDB-TF: HF-PCIC of 112 nm and PBDB-TF: PTIC of 109 nm) and the thickness of ~ 80 nm for neat acceptor films (IT-4F of 81nm, HF-PCIC of 83 nm and PTIC of 82 nm) under continuous one-sun equivalent illumination.

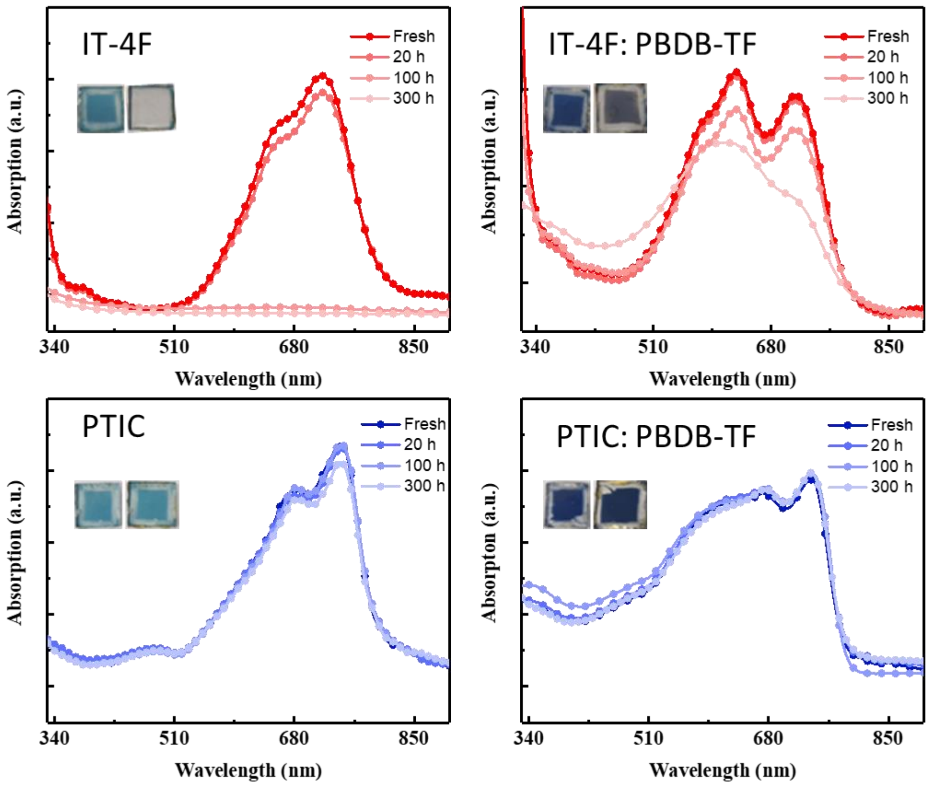

Supplementary Figure 4: UV–vis absorption spectra and photo images of encapsulated films (100 °C annealing for 10 minutes and then encapsulated with epoxy sealant in glovebox) under one-sun equivalent illumination in ambient.

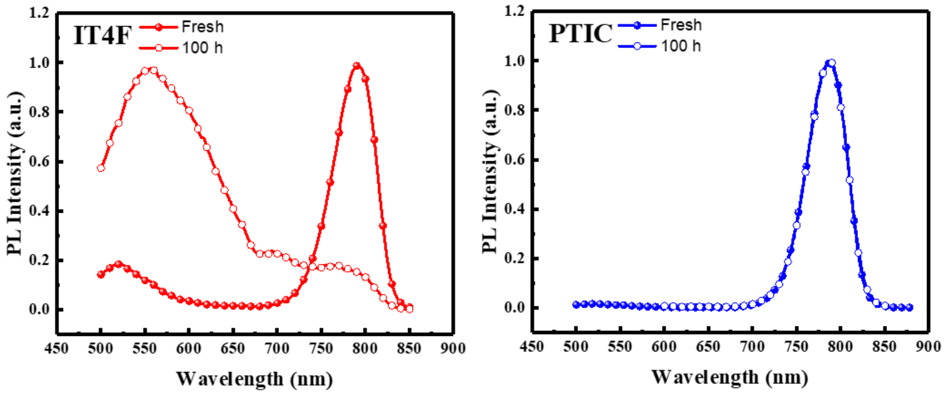

Supplementary Figure 5: Photoluminescence of encapsulated films before and after 100 hours one-sun equivalent illumination in ambient.

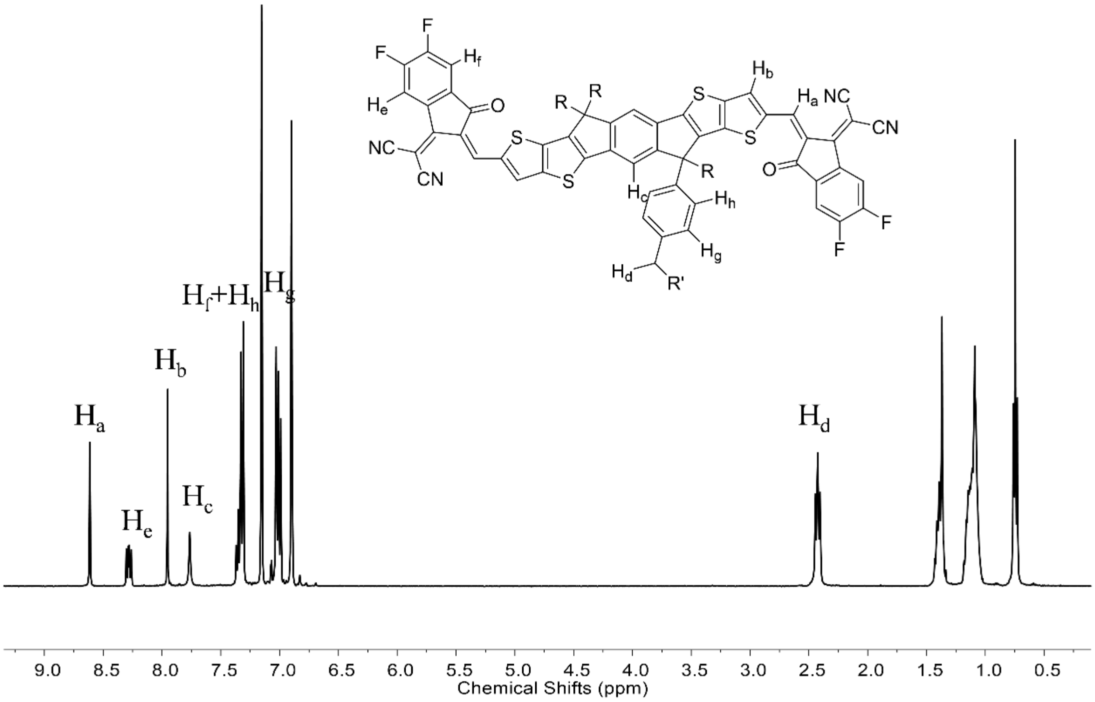

Supplementary Figure 6: The ^{1}H NMR of IT-4F.

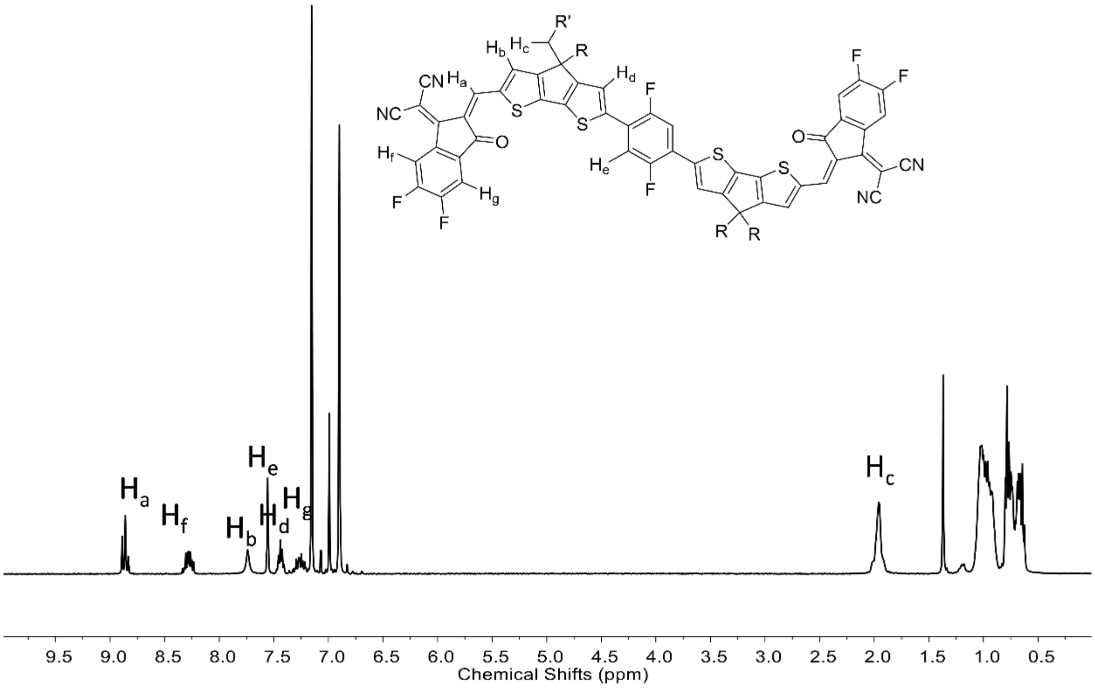

Supplementary Figure 7: The ^{1}H NMR of HF-PCIC.

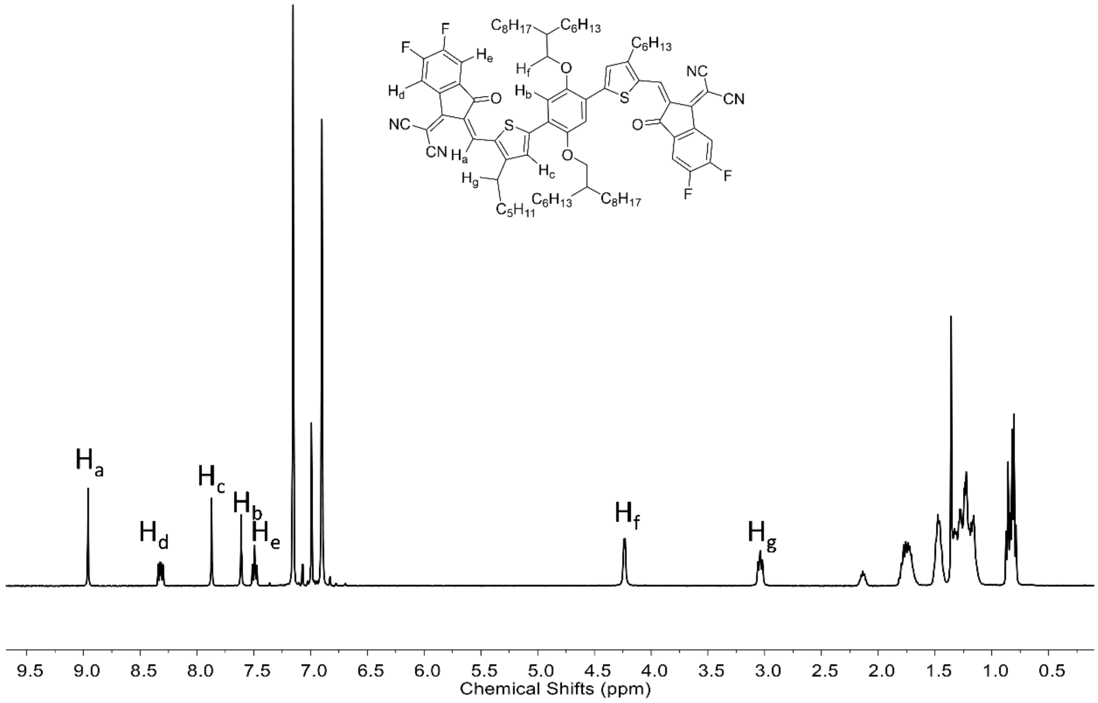

Supplementary Figure 8: The ^{1}H NMR of PTIC.

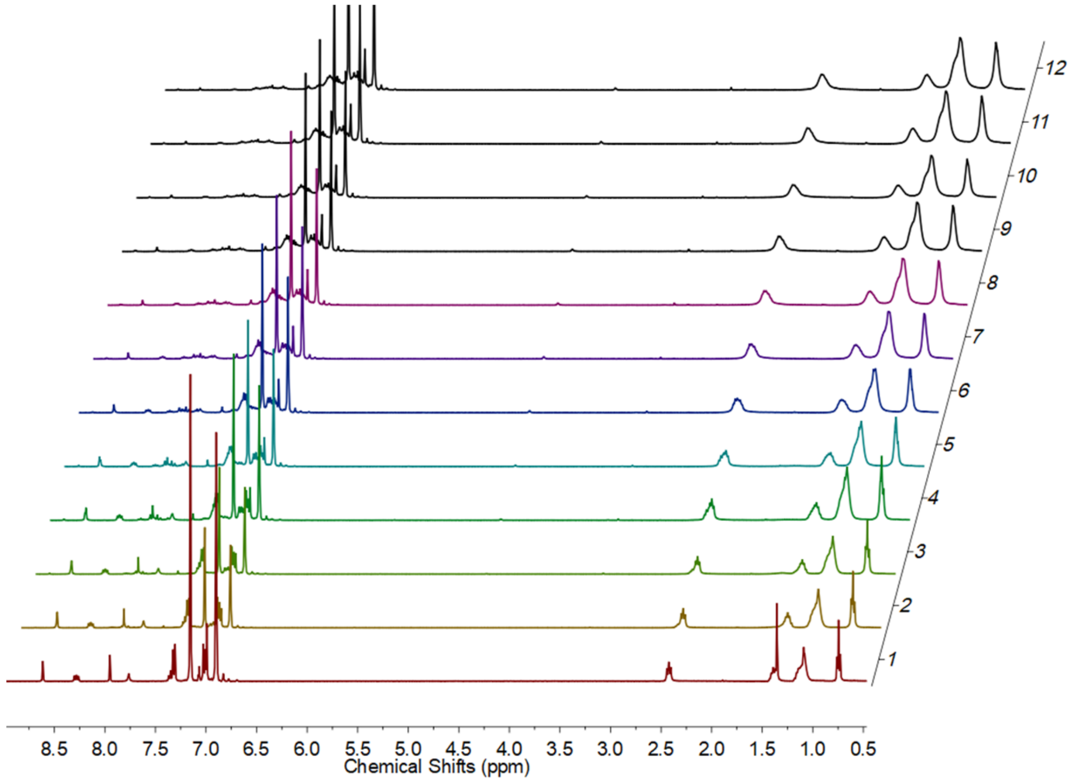

Supplementary Figure 9: The ^{1}H NMR of IT-4F under continuous one-sun equivalent illumination in solution. Line 1-12 present spectra were recorded at different time (Fresh, 10 h, 20 h, 30 h, 40 h, 60 h, 80 h, 100 h, 130 h, 160 h, 190 h and 220 h).

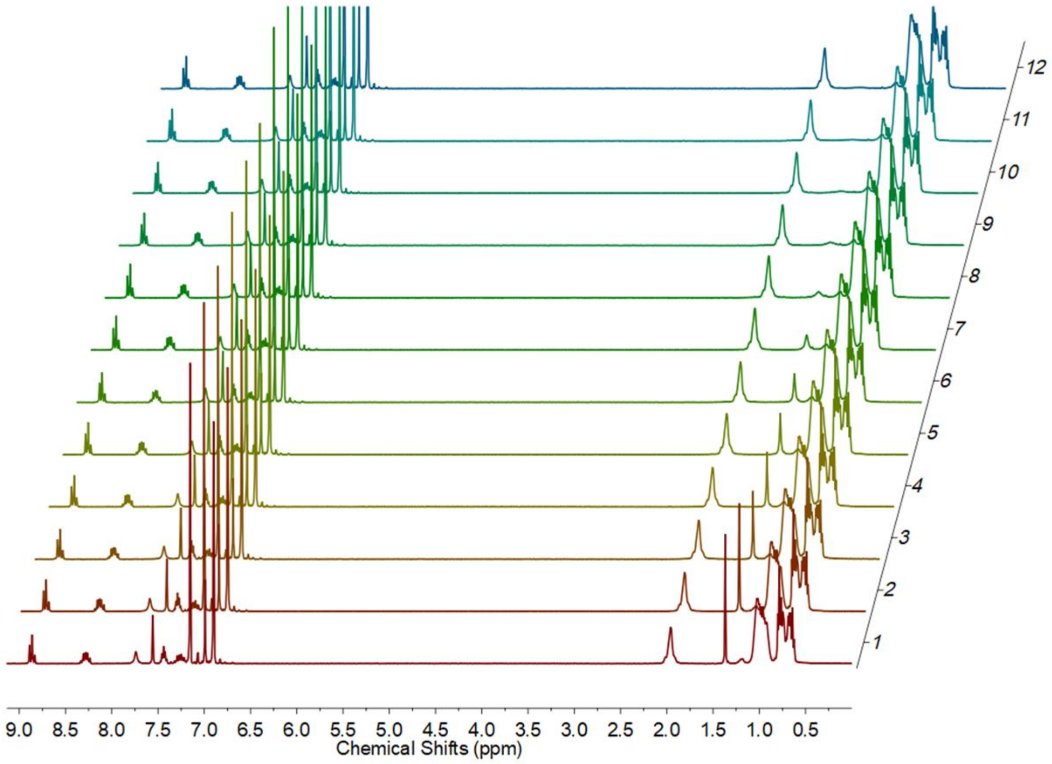

Supplementary Figure 10: The ^{1}H NMR of HF-PCIC under continuous one-sun equivalent illumination in solution. Line 1-12 present spectra were recorded at different time (Fresh, 10 h, 20 h, 30 h, 40 h, 60 h, 80 h, 100 h, 130 h, 160 h, 190 h and 220 h).

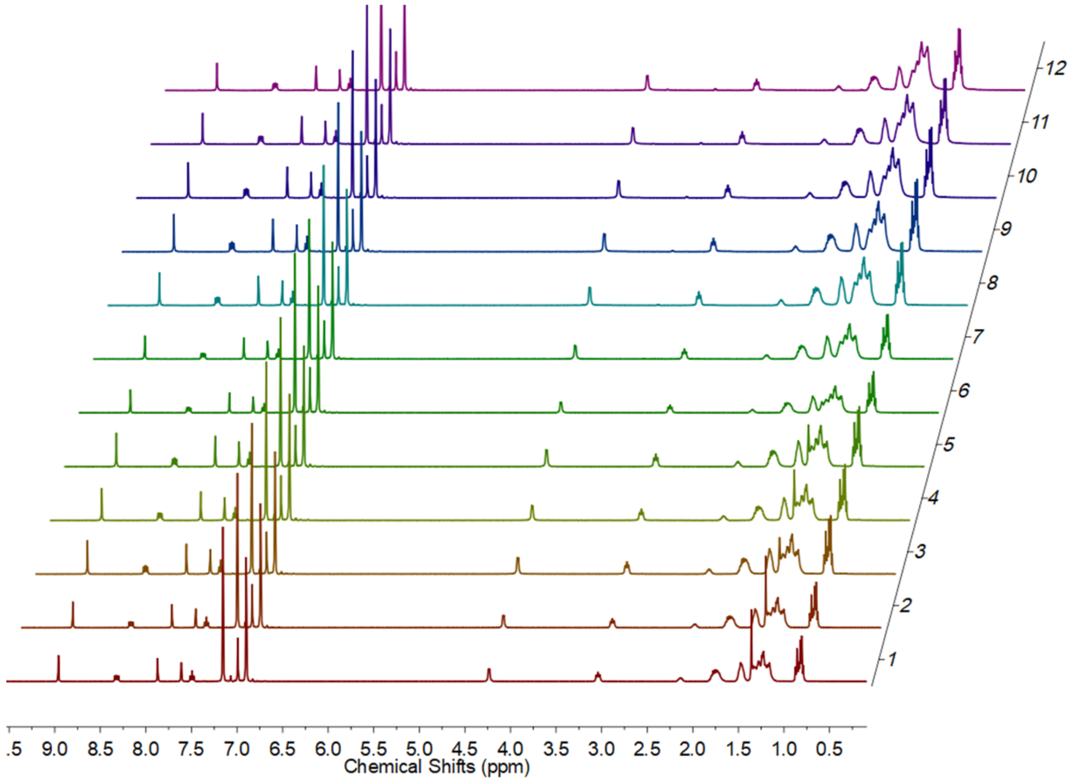

Supplementary Figure 11: The ^{1}H NMR of PTIC under continuous one-sun equivalent illumination in solution. Line 1-12 present spectra were recorded at different time (Fresh, 10 h, 20 h, 30 h, 40 h, 60 h, 80 h, 100 h, 130 h, 160 h, 190 h and 220 h).

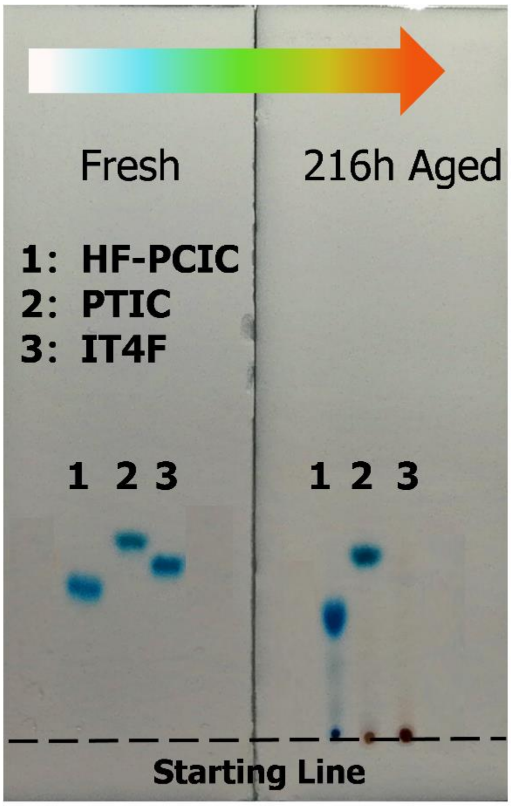

Supplementary Figure 12: The thin layer chromatography (TLC) of HF-PCIC, PTIC and IT-4F solution before and after 216 hours continuous one-sun equivalent illumination.

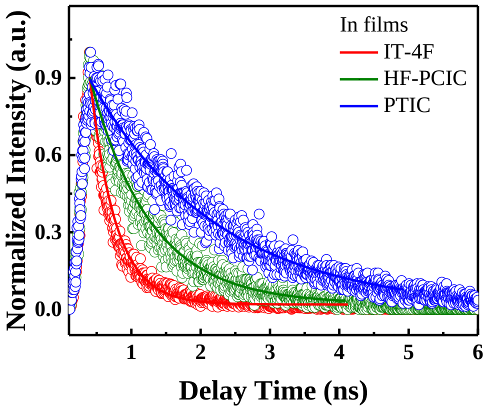

Supplementary Figure 13: Time-resolved photoluminescence for IT-4F, HF-PCIC and PTIC in films.

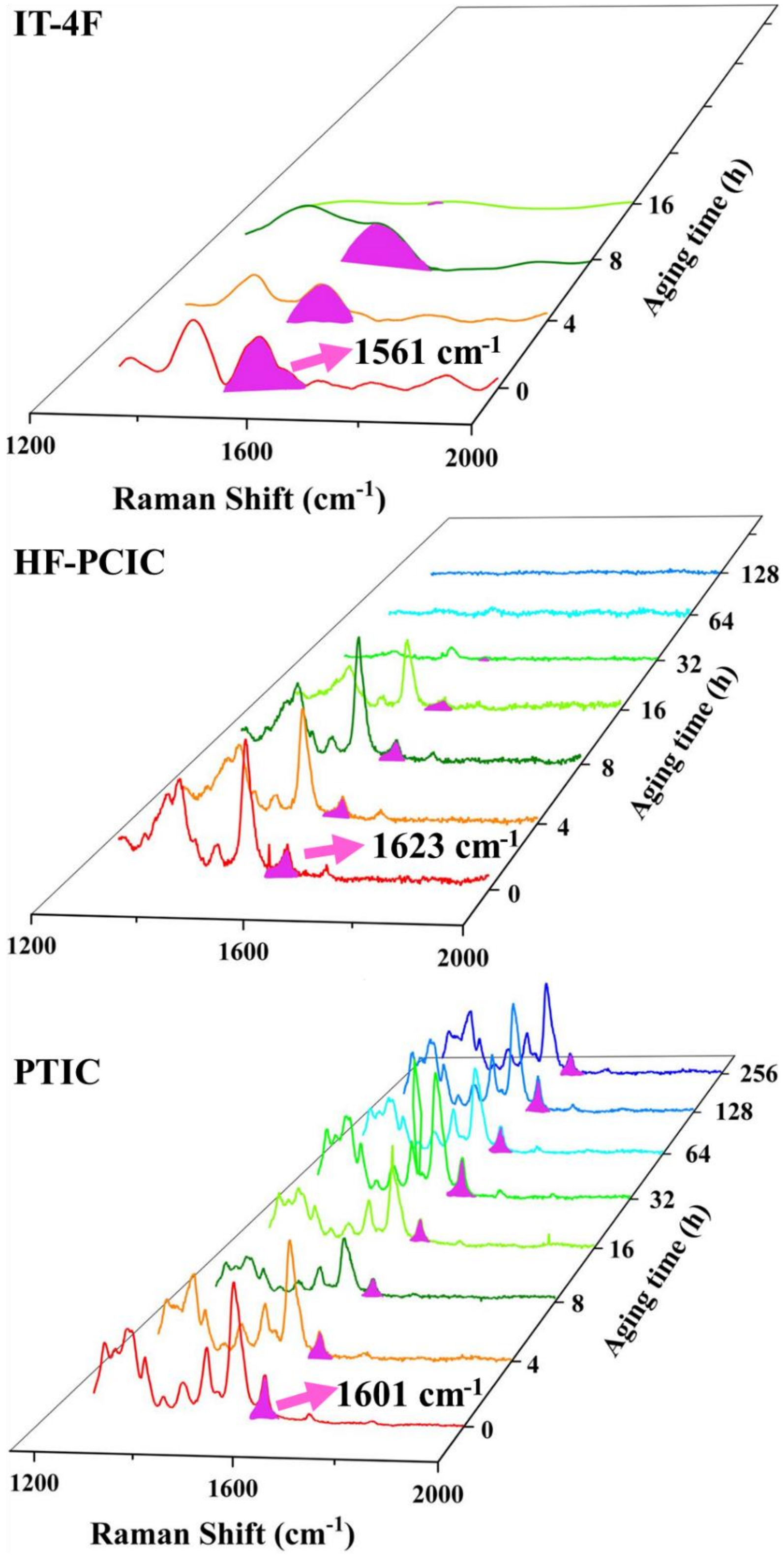

Supplementary Figure 14: Enlarged Raman spectrum of NFA films under continuous one-sun equivalent illumination. The highlighted purple peaks represent the Raman signals of vinyl double bond (IT-4F 1561 cm^{-1}, HF-PCIC 1561 cm^{-1}, PTIC 1601 cm^{-1}).

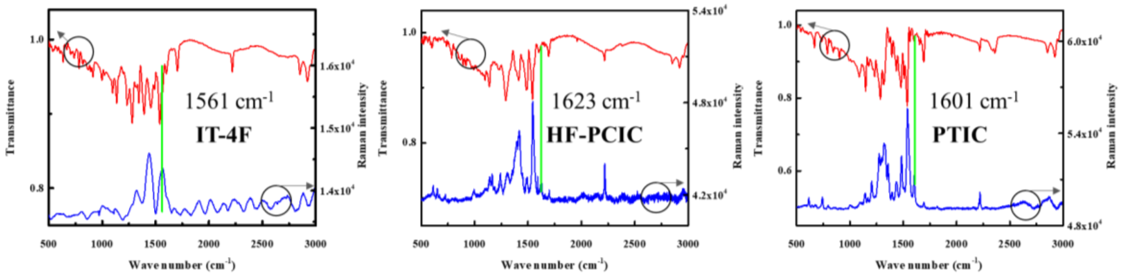

Supplementary Figure 15: Raman spectrum (blue curves) and IR spectrum (red curves) of NFAs, The marked peaks by green line represent the signals of exocyclic double bond (IT-4F 1561 cm^{-1}, HF-PCIC 1561 cm^{-1}, PTIC 1601 cm^{-1}).

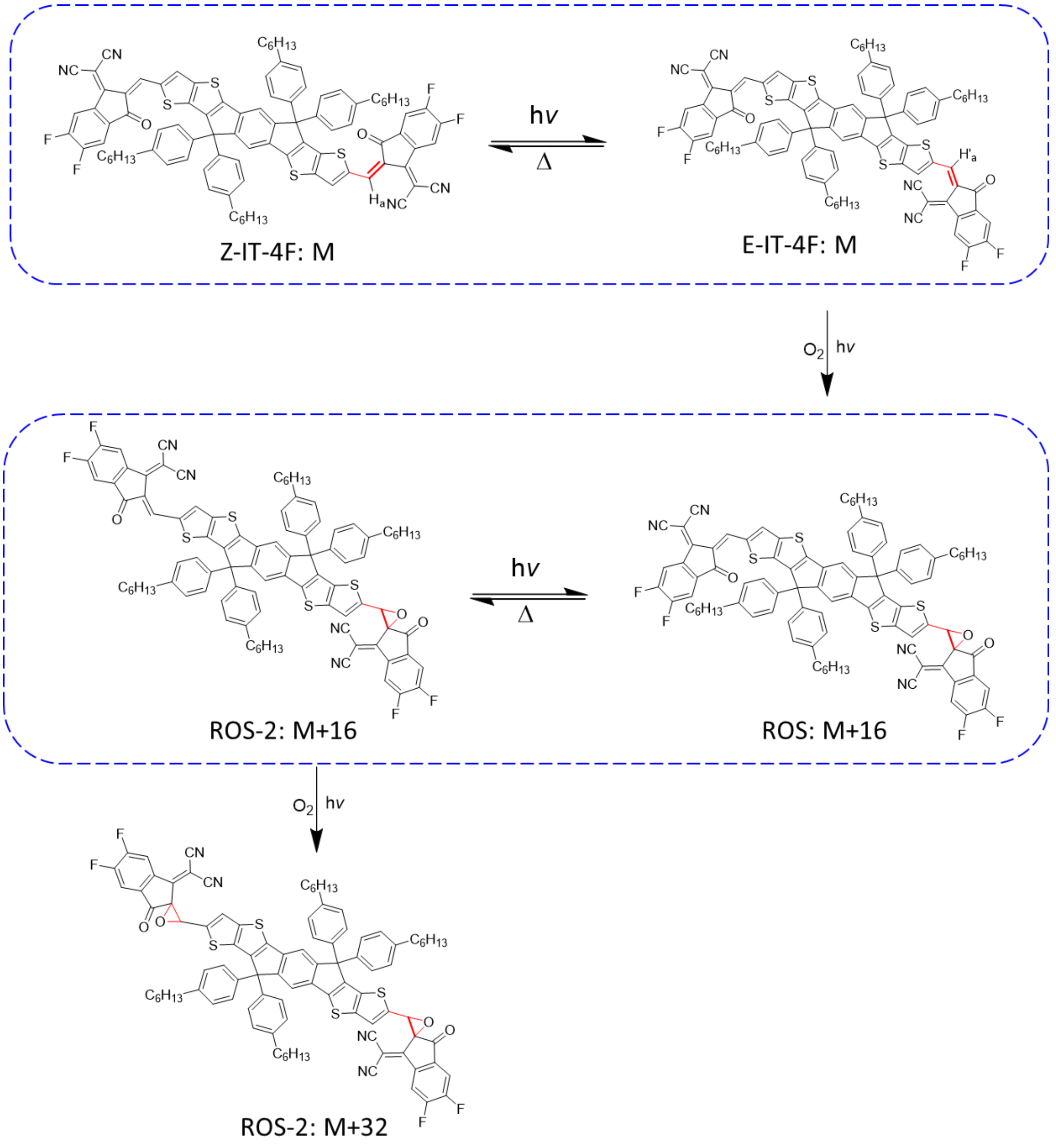

Supplementary Figure 16: The plausible photodegradation of IT-4F under one-sun equivalent illumination in ambient conditions.

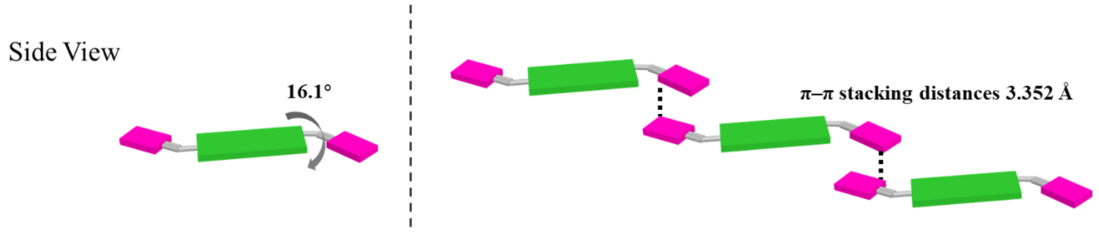

Supplementary Figure 17: Schematic illustration of IT-4F stacking in crystal solid according to the reported single crystal data.[1]

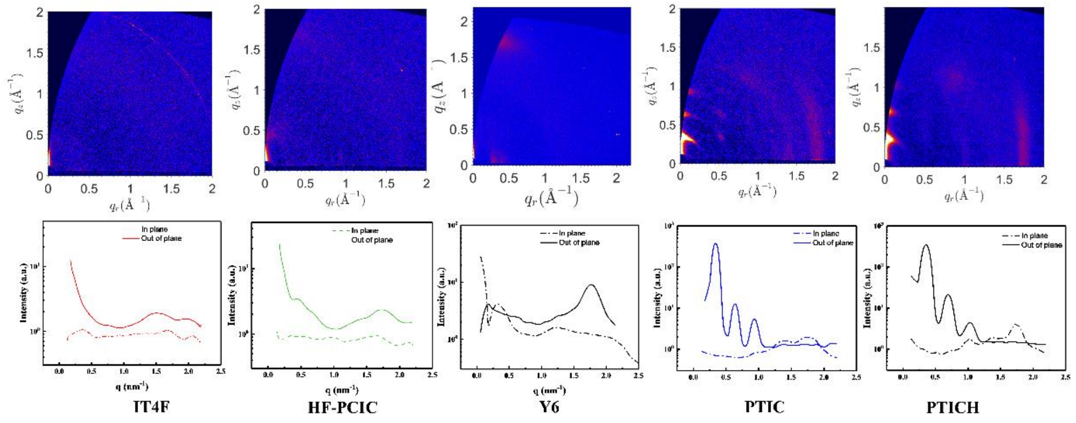

Supplementary Figure 18: The 2D GIWAXS images and 1D X-ray profiles along the q_z (out of plane) axis and q_r (in plane) axis of the corresponding NFA thin films.

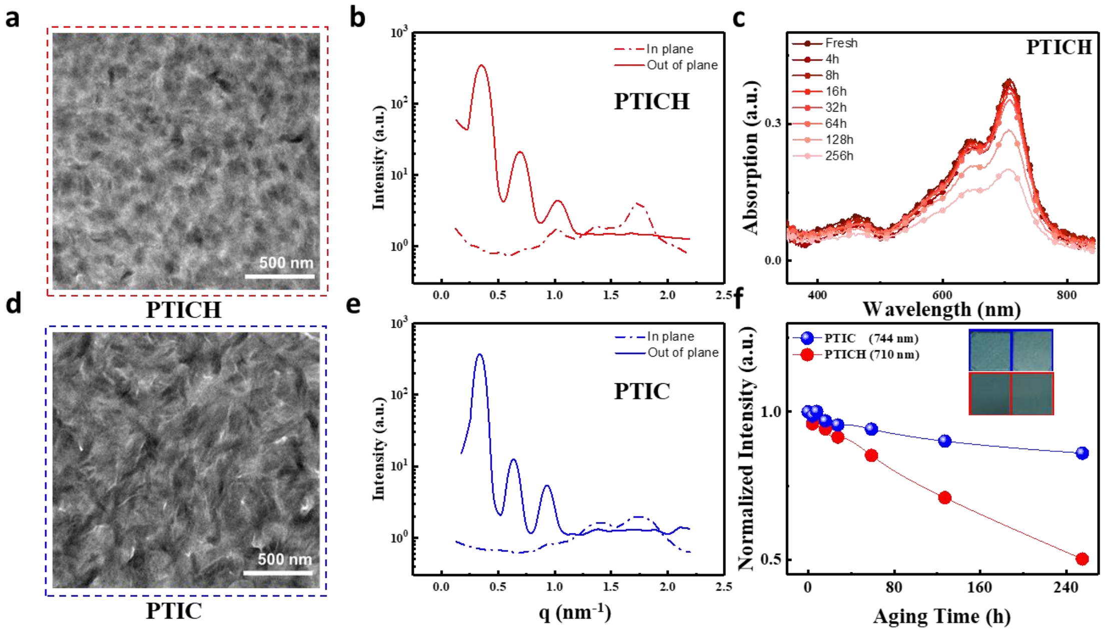

Supplementary Figure 19: The transmission electron microscopy (TEM) images of **a**, PTICH (w/o sidechain) and **d**, PTIC (with C_6H_{13} sidechain); **b**, and **e**, 1D X-ray profiles along the q_z (out of plane) axis and q_r (in plane) axis of PTICH and PTIC. **c**, and **f**, UV–vis absorption spectra and photo images of films under continuous one-sun equivalent illumination in ambient conditions.

Supplementary Table 1: The locations of π–π stacking peaks, π–π stacking distances, the calculated full width at half-maximum (FWHM), lamellar stacking peaks and lamellar stacking distances of neat NFAs films.

Materials	π–π stacking locations (Å^{-1})	FWHM (Å^{-1})	π–π stacking distances (Å)	Lamellar stacking locations (Å^{-1})	Lamellar stacking distances (Å)
IT-4F	1.61	0.27	3.90	0.32	19.62
HF-PCIC	1.72	0.26	3.65	0.34	18.47
PTIC	1.76	0.20	3.57	0.34	18.47
PTICH	1.73	0.22	3.63	0.35	17.94
Y6	1.76	0.24	3.57	0.33	19.03

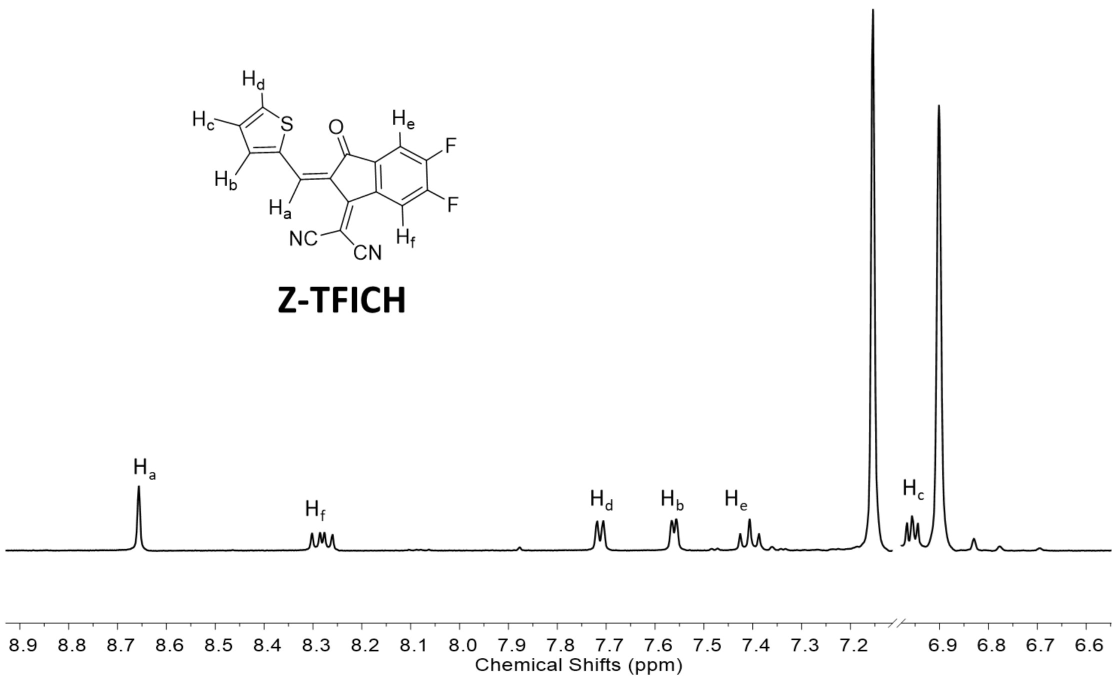

Supplementary Figure 20: The ^{1}H NMR of Z-TFICH.

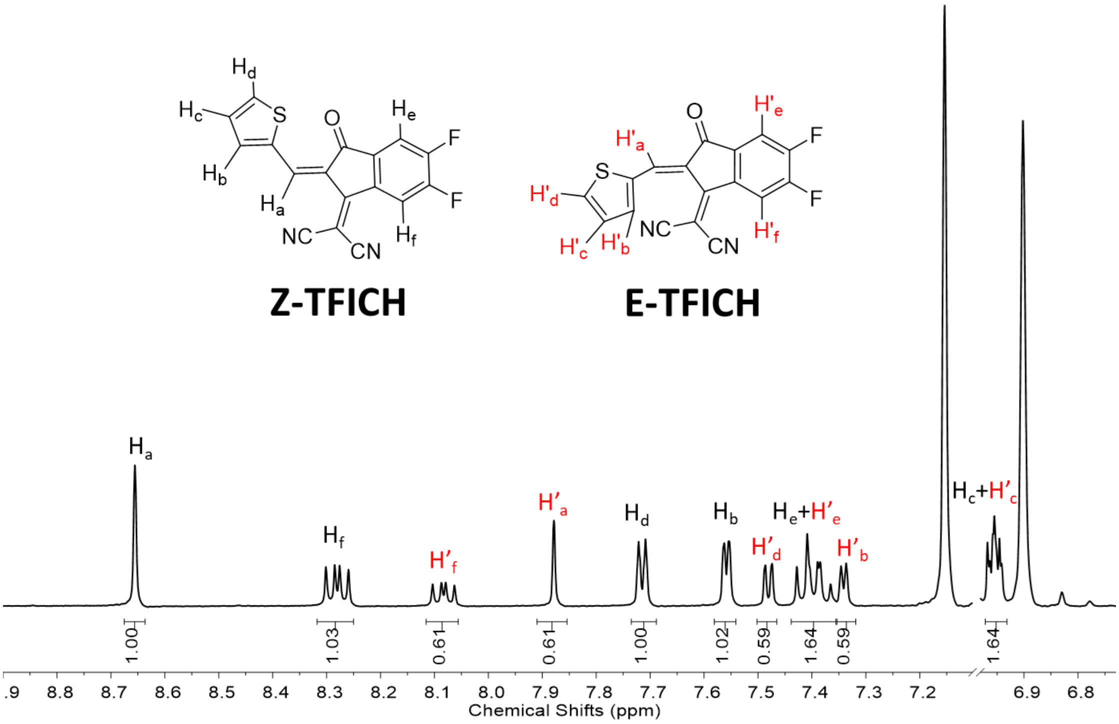

Supplementary Figure 21: The ^{1}H NMR of Z-TFICH in solution after 10 hours continuous one-sun equivalent illumination, with the generation of isomer co-mixture (Z-TFICH: E-TFICH 1:0.61).

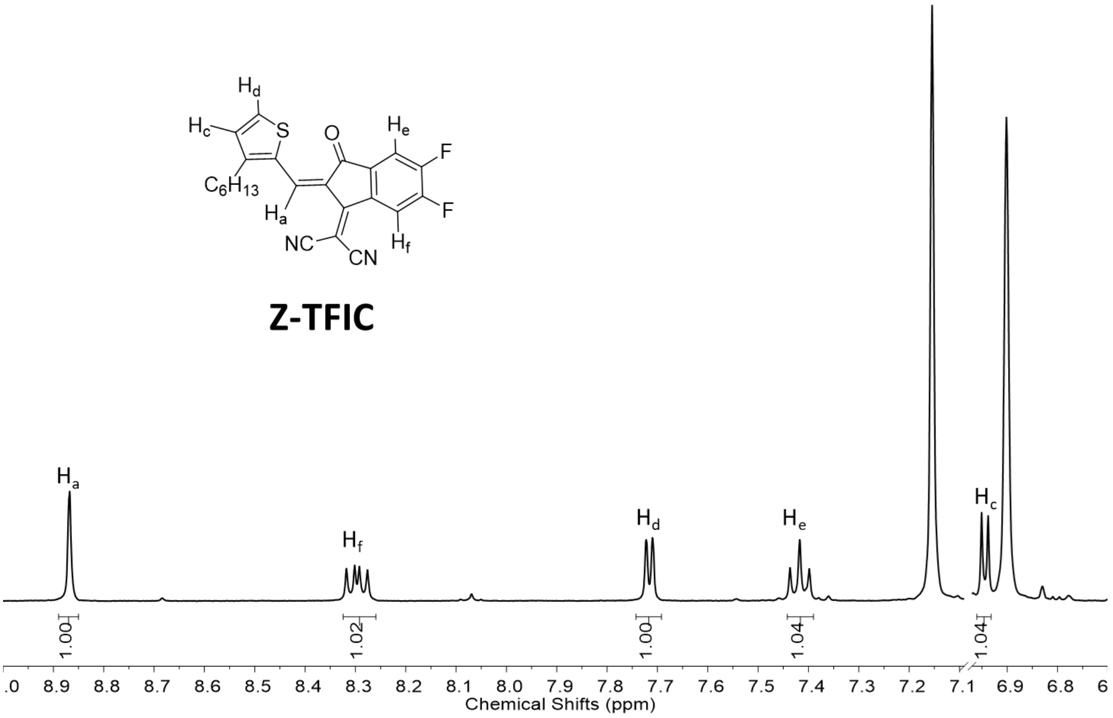

Supplementary Figure 22: The ^{1}H NMR of Z-TFIC.

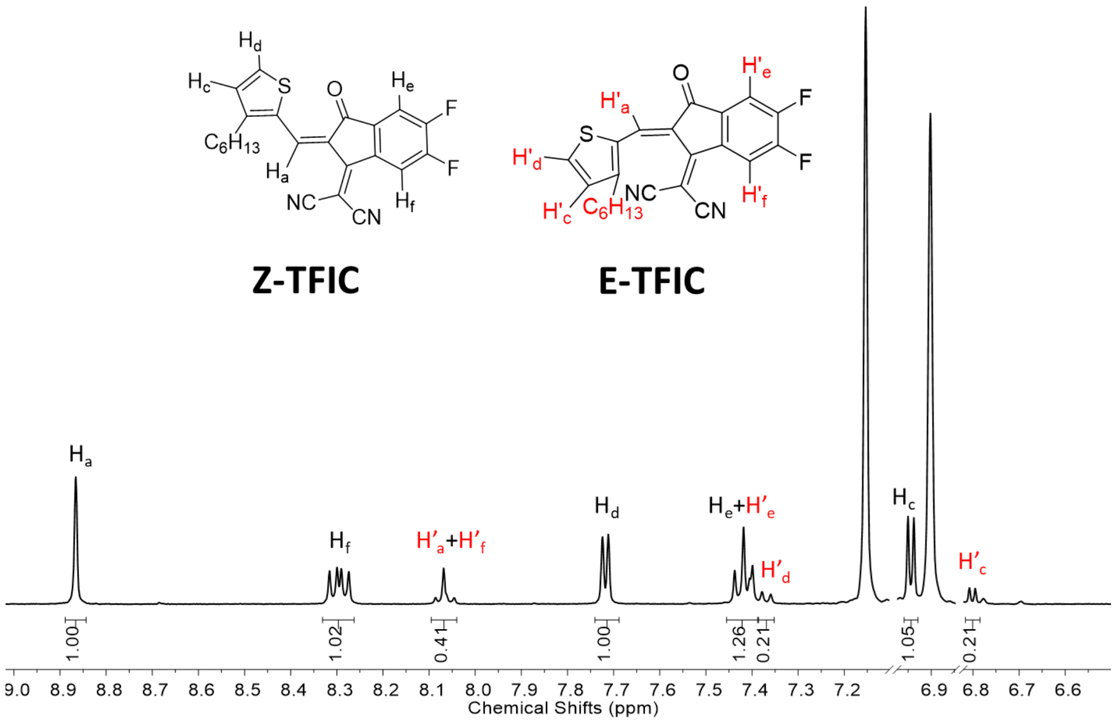

Supplementary Figure 23: The ^{1}H NMR of Z-TFIC in solution after 10 hours under continuous one-sun equivalent illumination, with the generation of isomer co-mixture (Z-TFIC: E-TFIC 1:0.21)

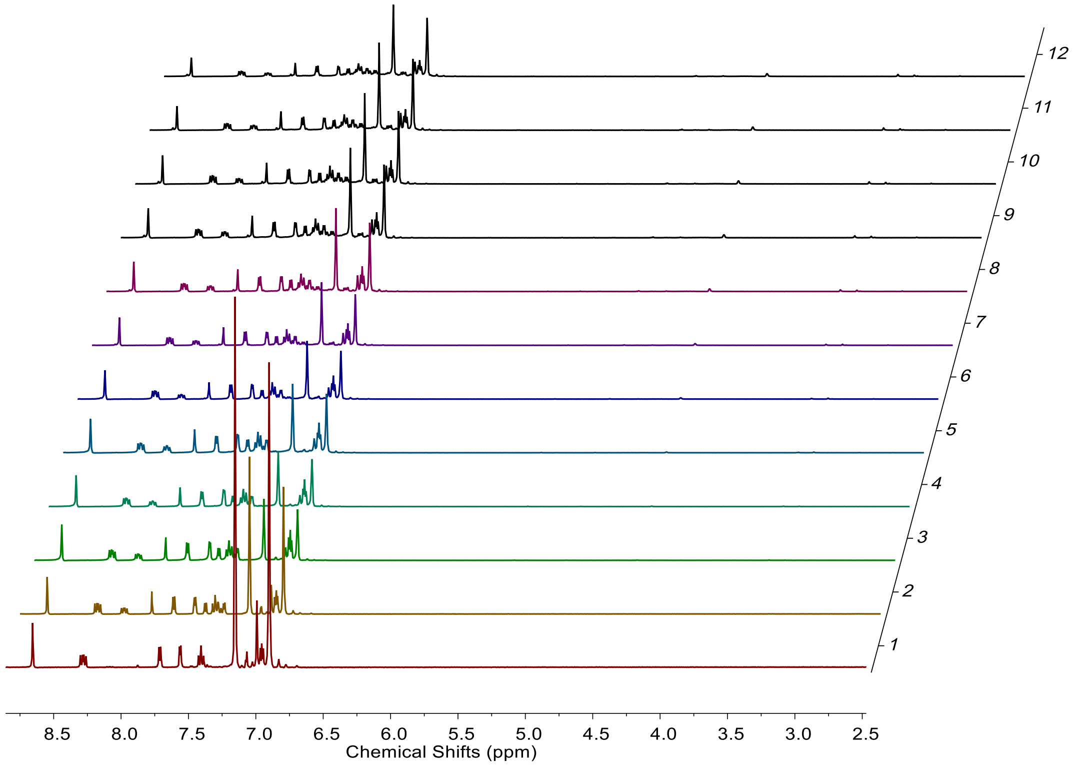

Supplementary Figure 24: The ^{1}H NMR of TFICH in solution under continuous one-sun equivalent illumination. Line 1-12 present spectra were recorded at different time

(Fresh, 10 h, 20 h, 30 h, 40 h, 60 h, 80 h, 100 h, 130 h, 160 h, 190 h and 220 h)

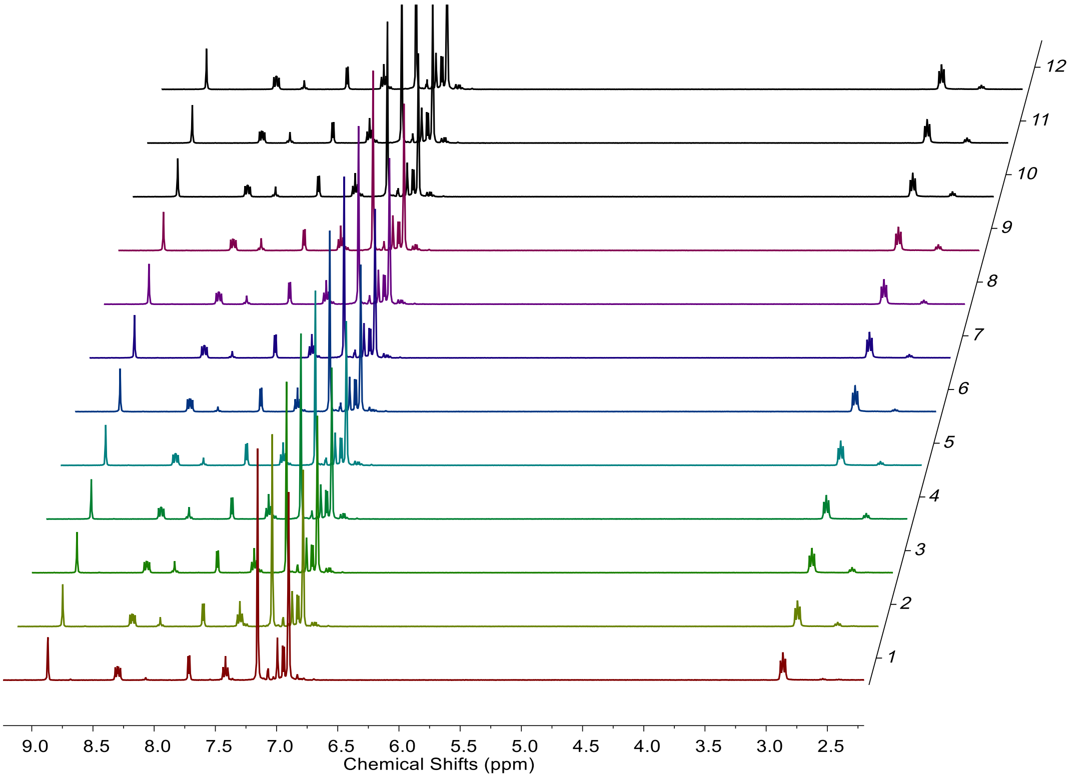

Supplementary Figure 25: The ^1H NMR of TFIC in solution under continuous one-sun equivalent illumination. Line 1-12 present spectra were recorded at different time (Fresh, 10 h, 20 h, 30 h, 40 h, 60 h, 80 h, 100 h, 130 h, 160 h, 190 h and 220 h)

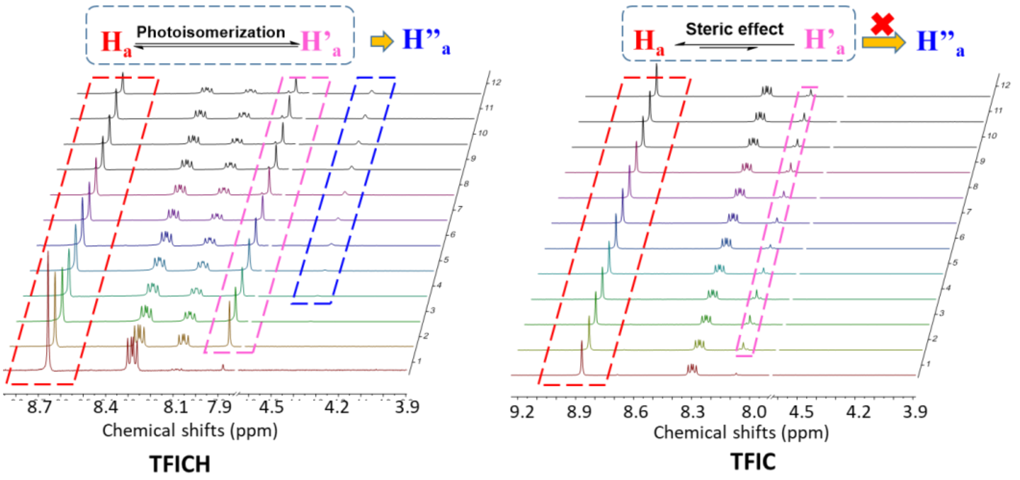

Supplementary Figure 26: The comparison of ^1H NMR of TFICH and TFIC under continuous one-sun equivalent illumination.

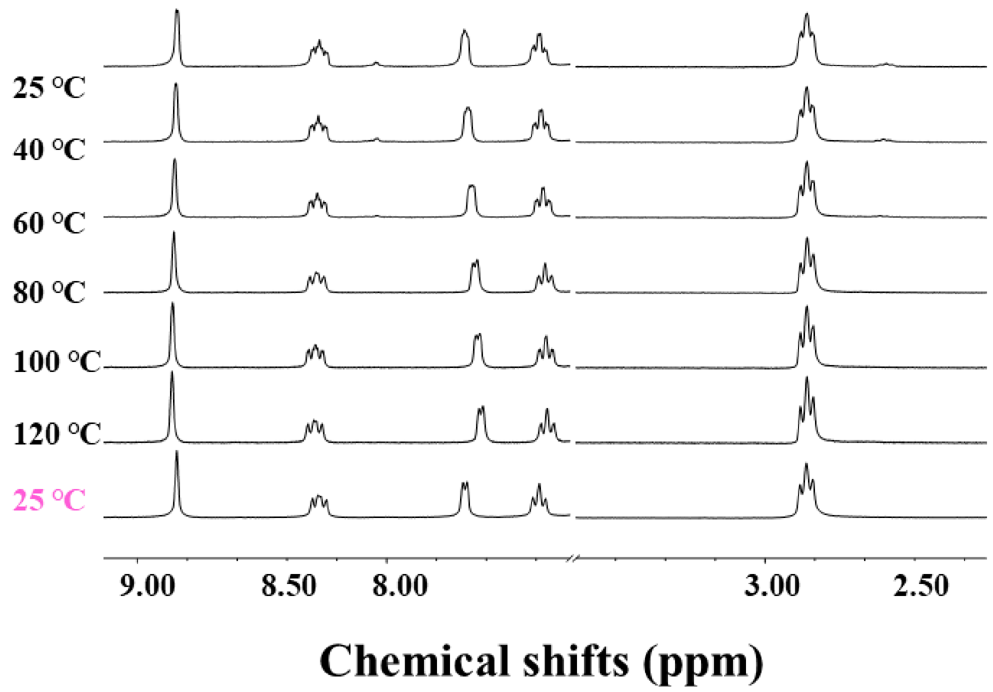

Supplementary Figure 27: The temperature-dependent ^{1}H NMR of TFIC-ES and TFIC-IS in d_4-$C_6D_4Cl_2$ (heated from 25-120 °C and then cool down to 25 °C)

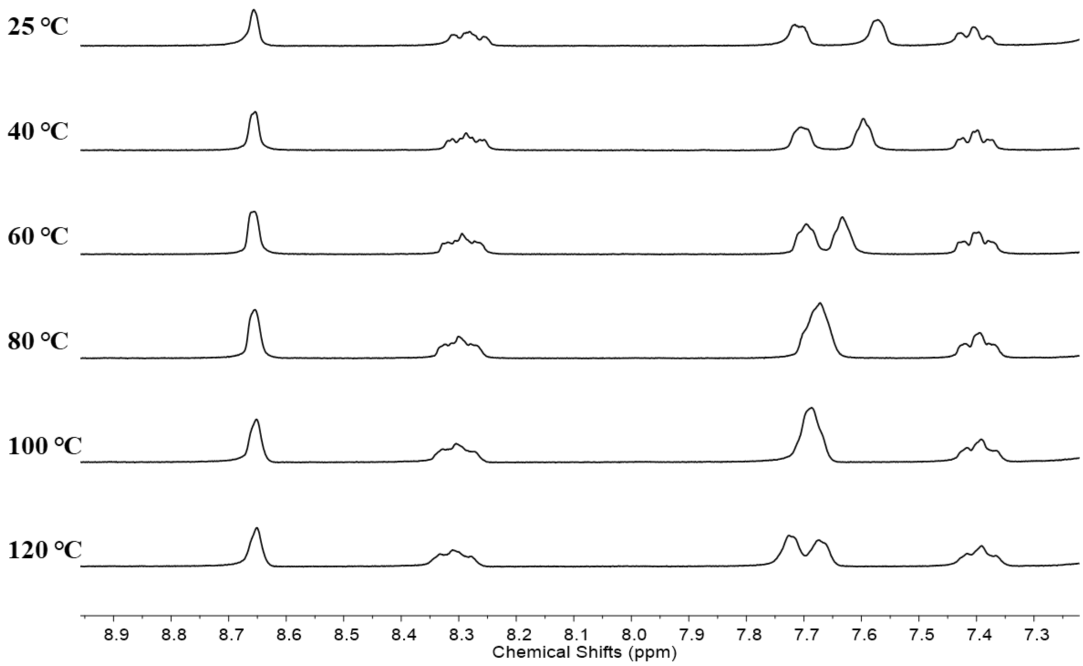

Supplementary Figure 28: The temperature-dependent ^{1}H NMR of TFICH in d_4-$C_6D_4Cl_2$ (heated from 25-120 °C)

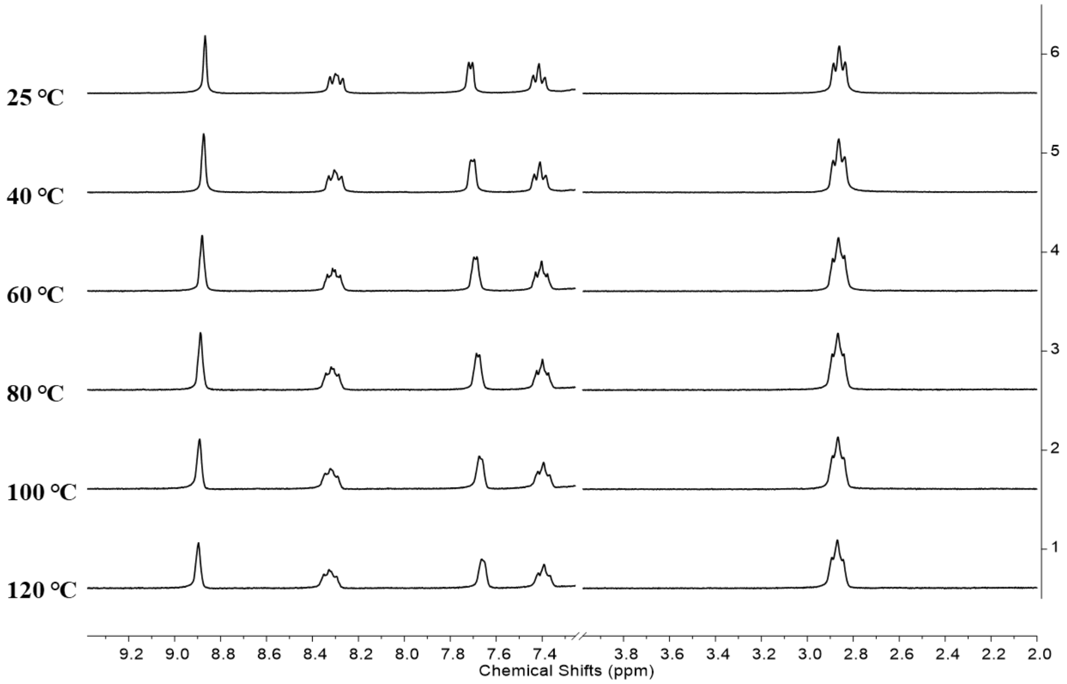

Supplementary Figure 29: The temperature-dependent ^{1}H NMR of TFIC in d_4-$C_6D_4Cl_2$ (heated from 25-120 °C)

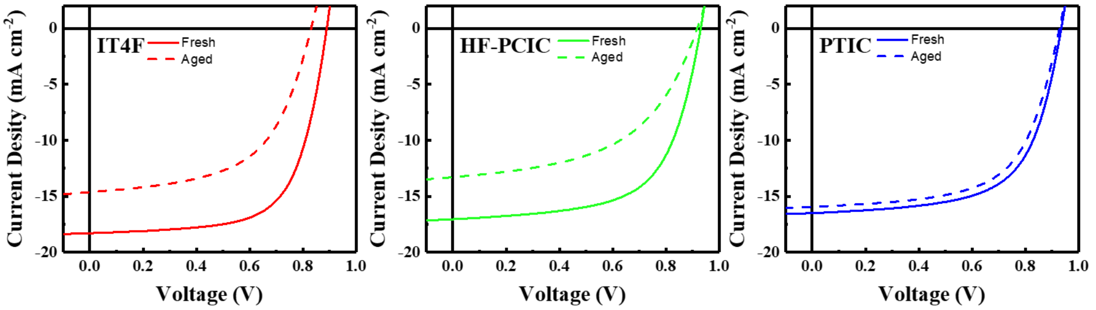

Supplementary Figure 30: *J-V* curves of the fresh and 50 hours illuminated devices based on IT-4F, HF-PCIC and PTIC, respectively.

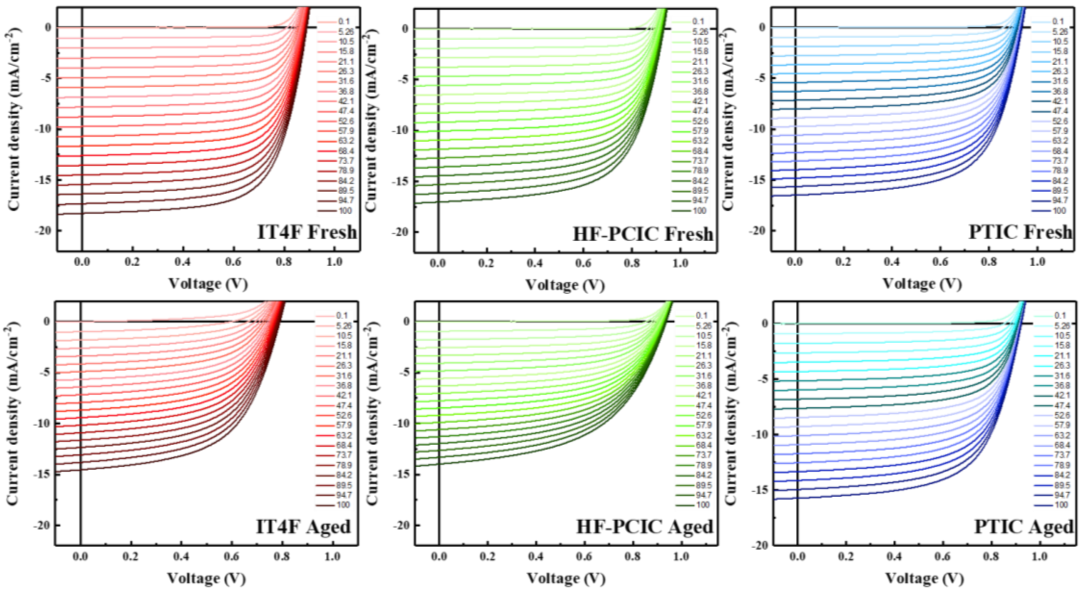

Supplementary Figure 31: *J–V* curves under the varied incident light intensity of the fresh and 50 hours illuminated devices based on IT-4F, HF-PCIC and PTIC, respectively.

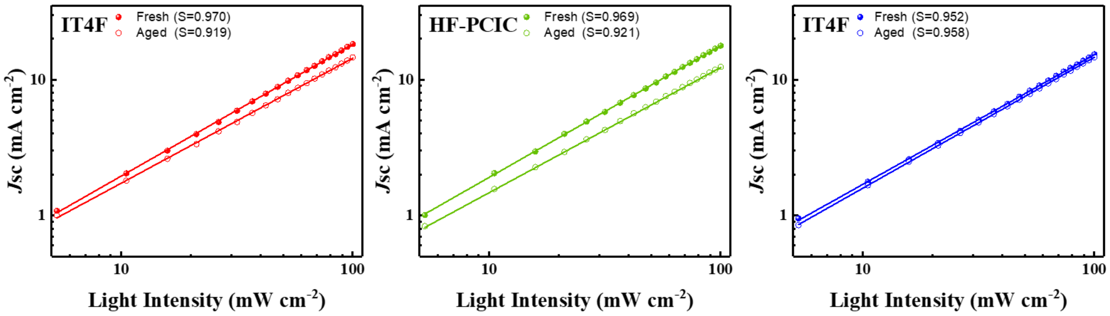

Supplementary Figure 32. Dependencies of J_{sc} on the varied light intensity of the fresh and 50 hours illuminated devices based on IT-4F, HF-PCIC and PTIC, respectively.

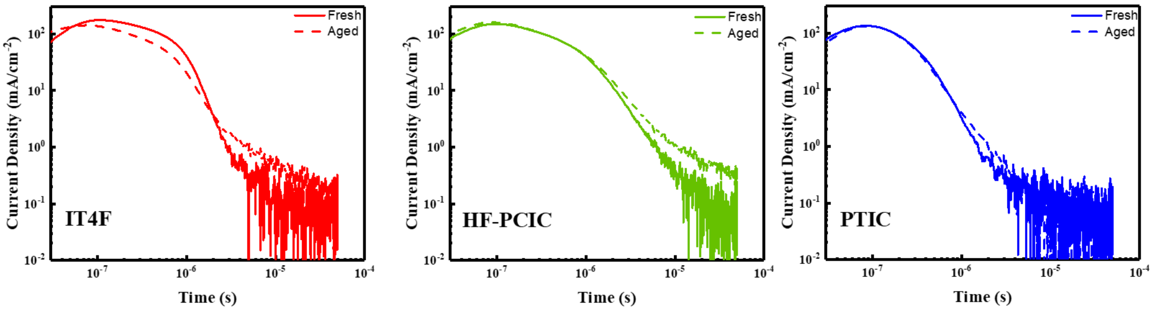

Supplementary Figure 33: Charge extraction curves of fresh (solid line) and 50 hours illuminated devices based on IT-4F, HF-PCIC and PTIC, respectively.

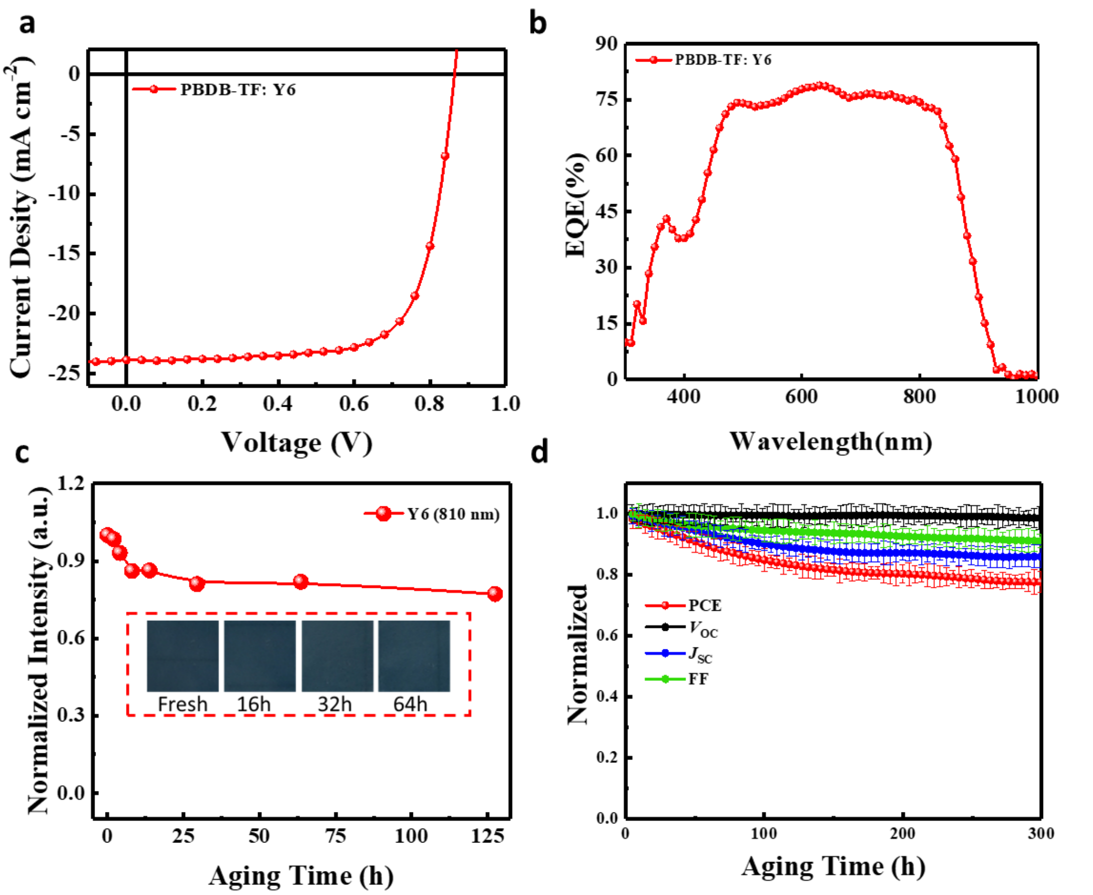

Supplementary Figure 34: **a**, The *J-V* and **b**, EQE curves of PBDB-TF:Y6 based OSCs; **c**, The change of absorption intensity at 810 nm and photo images of Y6 films under illumination **d**, Stabilities of encapsulated PBDB-TF:Y6 based OSCs without any solvent additive (fresh OSCs with decent parameter: V_{oc} 0.86 V, J_{sc} 23.84 mA/cm^{-2}, FF 0.72, and PCE 14.76 %) under continuous one-sun equivalent illumination.

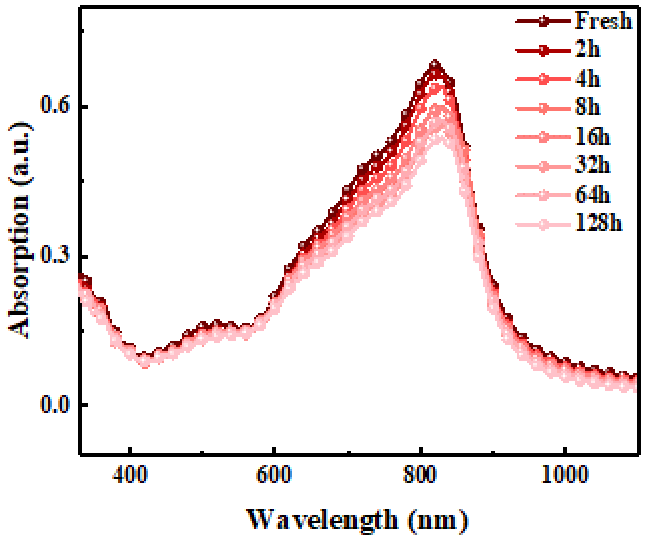

Supplementary Figure 35: UV–vis absorption spectra of Y6 films under continuous one-sun equivalent illumination in ambient conditions.

Supplementary Table 2: Photovoltaic parameters of the OSCs based on PBDB-TF: IT-4F, PBDB-TF: HF-PCIC and PBDB-TF: PTIC blends without any solvent additive.

Acceptors	V_{oc} (V)	J_{sc} (mA/cm^2)	FF (%)	PCE (%)
IT-4F	0.87 (0.86±0.01)	18.27 (18.01±0.42)	0.70 (0.68±0.03)	11.13 (10.77±0.36)
HF-PCIC	0.91 (0.90±0.01)	17.00 (16.72±0.37)	0.65 (0.62±0.03)	10.06 (9.73±0.33)
PTIC	0.93 (0.92±0.01)	16.49 (16.32±0.25)	0.67 (0.66±0.02)	10.28 (10.07±0.21)

Average PCEs for 20 devices in brackets.

Preparation of TFICH.

The thiophene-2-carbaldehyde (112 mg, 1.0 mmol), 2-(5,6-difluoro-3-oxo-2,3-dihydro-1H-inden-1-ylidene) malononitrile (250 mg, 1.1 mmol) and a few drops of pyridine were dissolved into chloroform. The mixture was then refluxed overnight. The solution was concentrated in vacuum. The crude product was washed with methanol and acetone, and then recrystallized with $CHCl_3$ and methanol. The crystalline solid was washed with methanol. ^{1}H NMR (400 MHz, $CDCl_3$) δ: 8.95 (s, 1H), 8.57 (dd, 1H, J = 6.5 Hz, 9.9 Hz), 8.03 (d, 1H, J = 4.8 Hz), 7.94 (d, 1H, J = 4.2 Hz), 7.73 (t, 1H, J = 7.6 Hz), 7.30 (dd, 1H, J = 3.9 Hz, 5.0 Hz). ^{13}C-NMR ($CDCl_3$, 100 MHz) δ: 185.58, 158.44, 145.00, 142.07, 138.64, 136.88, 128.83, 122.92, 115.21, 114.99, 113.80, 113.57, 113.08, 112.87.

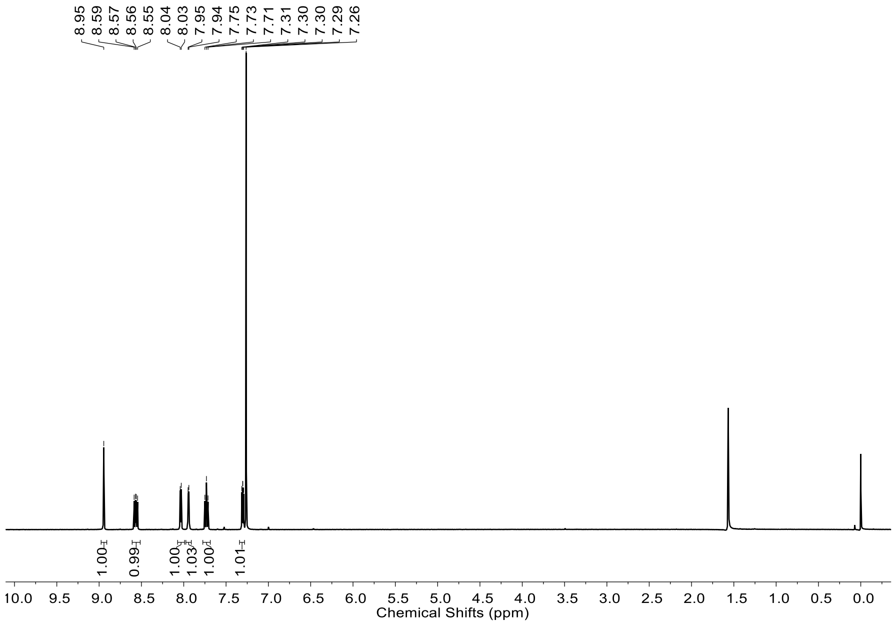

^{1}H NMR of TFICH

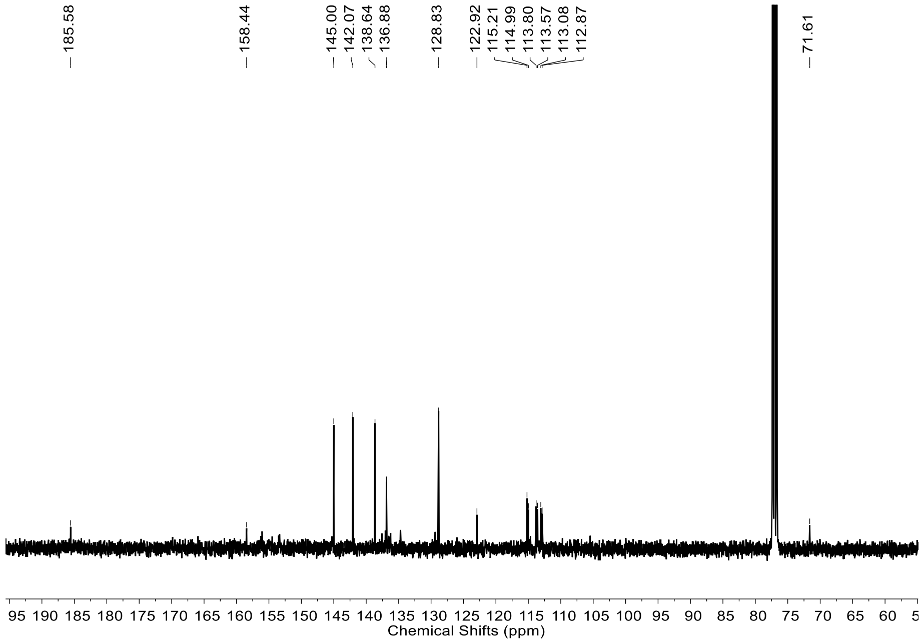

^{13}C NMR of TFICH

Preparation of TFIC.

The 3-hexylthiophene-2-carbaldehyde (196 mg, 1.0 mmol), 2-(5,6-difluoro-3-oxo-2,3-dihydro-1H-inden-1-ylidene)malononitrile (250 mg, 1.1 mmol) and a few drops of pyridine were dissolved into chloroform. The mixture was then refluxed overnight. The solution was concentrated in vacuum. The crude products were purified by column chromatography (SiO_2, petroleum ether/ dichloromethane = 1/1). The yellow solid was washed with methanol and acetone, then recrystallized by $CHCl_3$ and methanol. The crystalline solid was washed with methanol. ^{1}H-NMR ($CDCl_3$, 400 MHz) δ: 9.09 (s, 1H), 8.60–8.55 (m, 1H), 7.96–7.95 (m, 1H, J = 5.2 Hz), 7.73-7.69 (m, 1H), 7.17-7.16 (d, 1H, J = 5.2 Hz), 3.01–2.97 (t, 2H, J = 7.8 Hz), 1.70–1.63 (m, 2H), 1.41–1.38 (m, 2H), 1.32–1.30 (m, 4H), 0.90-0.87 (t, 3H, J = 7.0 Hz). ^{13}C-NMR ($CDCl_3$, 100 MHz) δ: ^{13}C NMR (101 MHz, CDCl3) δ 185.68, 161.14, 159.26, 140.84, 135.95, 131.72, 130.66, 121.40, 115.11, 114.88, 114.28, 114.12, 112.88, 112.69, 70.48, 31.96, 31.59, 30.14, 29.06, 22.53, 14.06.

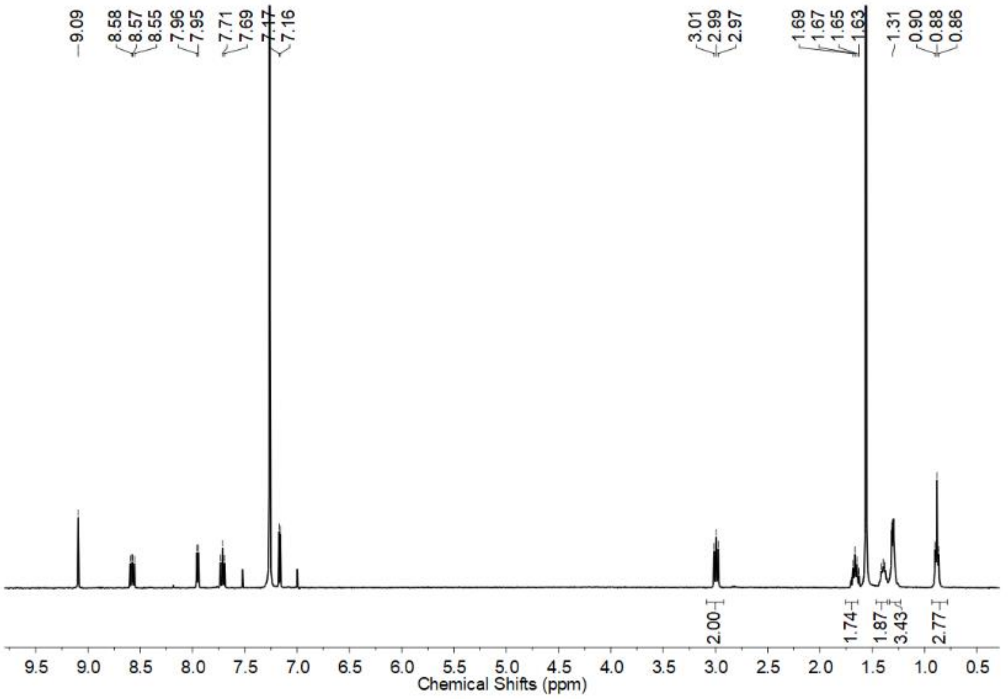

^{1}H NMR of TFIC

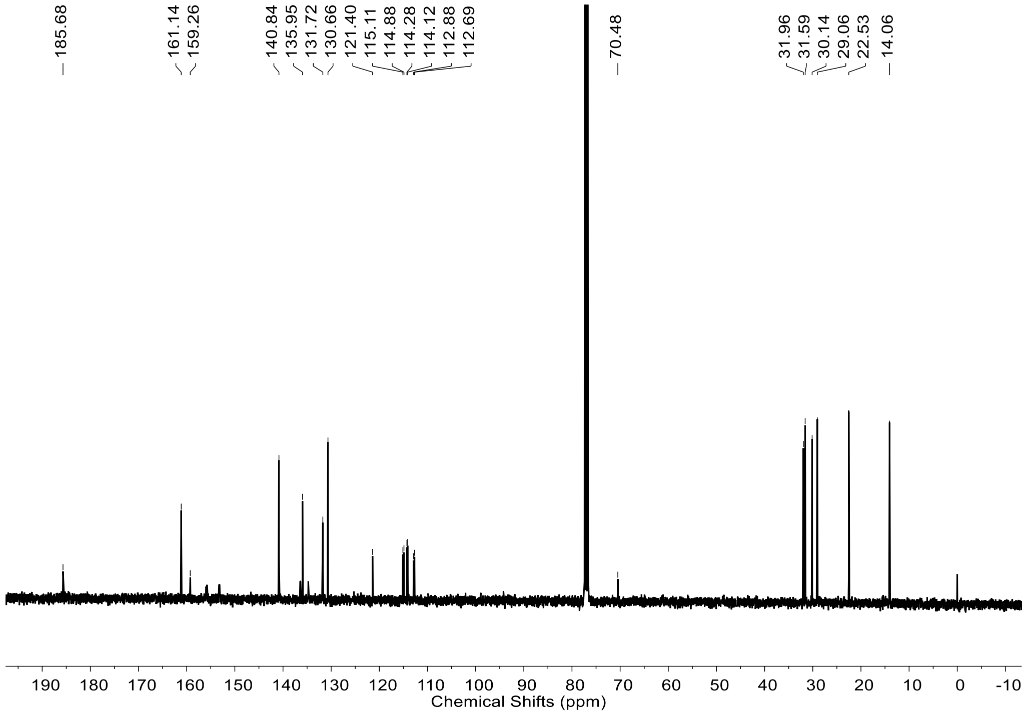

^{13}C NMR of TFIC

Reference

1. Aldrich TJ, Matta M, Zhu W, Swick SM, Stern CL, Schatz GC, *et al.* Fluorination Effects on

Indacenodithienothiophene Acceptor Packing and Electronic Structure, End-Group Redistribution, and Solar Cell Photovoltaic Response. *J. Am. Chem. Soc.* **141,** 3274-3287 (2019).
